# Complete mitochondrial genomes of two rockfish: *Sebastes maliger* and *Sebastes norvegicus* (Scorpaenidae, Scorpaeniformes)

**DOI:** 10.1080/23802359.2022.2116951

**Published:** 2022-09-07

**Authors:** Jillian R. Campbell, Peter C. Searle, Andrea L. Kokkonen, Dennis K. Shiozawa, Mark C. Belk, R. Paul Evans

**Affiliations:** a Department of Biology, Brigham Young University, Provo, UT, USA; b Department of Microbiology and Molecular Biology, Brigham Young University, Provo, UT, USA; c Monte L. Bean Life Science Museum, Brigham Young University, Provo, UT, USA

**Keywords:** *Sebastes maliger*, *Sebastes norvegicus*, mitogenome, rockfish

## Abstract

We report the complete mitochondrial genomes of two rockfish: *Sebastes maliger* and *Sebastes norvegicus*. The mitogenomes consist of 13 protein-coding regions, 22 tRNAs, two rRNAs, and one control region. *Sebastes* mitogenome control regions are highly variable due to the presence of repeat sequences. The mitogenomes for *S. maliger* and *S. norvegicus* are 16,403 and 16,401 bp, respectively. Using these two mitogenomes and 25 additional *Sebastes* mitogenomes from GenBank, we examine the phylogenetic relationships in *Sebastes*. *Sebastes maliger* is sister to a clade including *S. rubrivinctus, S. nigrocinctus, S. umbrosus,* and *S. oculatus*, while *S. norvegicus* is sister to *S. fasciatus*.

*Sebastes* (Cuvier, 1829) is a diverse genus of marine fish, comprising at least 110 species found mostly in the North Pacific, with a small number of species in the Atlantic, Arctic, and Indian Oceans (Kim and Lee 2004; Hyde and Vetter 2007). The Quillback rockfish, *Sebastes maliger* (Jordan and Gilbert, 1880), is found in the North-East Pacific Ocean (Munk 2001; Love et al. 2002; West et al. 2014); whereas the Golden redfish, *Sebastes norvegicus* (Ascanius, 1772), is found in the North Atlantic Ocean (Rolskii et al. 2020). Although previous studies have focused on the mitogenomes of Western Pacific rockfish, few studies have evaluated the mitogenomes of East Pacific and Atlantic species. We provide further resolution of the phylogenetic relationships in *Sebastes* by assembling the complete mitogenomes of *S. maliger* and *S. norvegicus.*

We collected *S. maliger* near Excursion Inlet, Alaska in the Gulf of Alaska (58.4854, −135.4521) using hook-and-line sampling (IACUC-approved protocol #15-0602). A liver sample was placed in RNAlater (MilliporeSigma, St. Louis, MO, USA), frozen at −20 °C, transported to Brigham Young University, and stored at −80 °C until processed. A morphological voucher was not retained. We extracted DNA and sequenced it using an Illumina HiSeq 2500 system (Illumina, San Diego California, USA) at Brigham Young University’s DNA Sequencing Center (Provo, Utah, USA). The tissue sample (267106) was deposited at the Monte L. Bean Life Science Museum, Brigham Young University (Jerald B. Johnson, jerry.johnson@byu.edu) and raw sequences were deposited in the SRA database (SRR15573079). We retrieved DNA sequences of *S. norvegicus* (ERR1473911) from the SRA database on NCBI (Malmstrøm et al. 2017). This specimen was collected near Ballstad in Lofoten, Norway (68.0713, 13.5361) by commercial fishermen (M. Malmstrøm, personal communication, November 29, 2021).

We checked sequence quality using FastQC (Andrews 2010). Mitogenomes were assembled with Geneious v. 11.0.9 (Biomatters Ltd., Auckland, New Zealand) by mapping short reads against three reference mitogenomes, *S. fasciatus*, *S. oculatus*, and *S. koreanus,* (KX897946, MN218776, KJ775792). We then extracted a single consensus contig for each assembly (>100x coverage for both mitogenomes for all assemblies). The mitogenomes were annotated using MitoAnnotator (Iwasaki et al. 2013). For our phylogenetic analysis, we acquired a total of 25 rockfish mitogenomes from GenBank. We generated multiple sequence alignments separately for each of the 13 protein-coding genes using MAFFT v. 7.475 (Katoh and Standley 2013) before concatenating them into a final file that was 11,412 bp in length after all positions with missing data were removed.

The complete mitogenomes of *S. maliger* and *S. norvegicus*, assembled against *S. fasciatus* (KX897946), were 16,403 and 16,401 bp in length, respectively (MW846222, MW846223). Depending on which reference mitogenome was used during assembly (KX897946, MN218776, KJ775792), the complete mitogenomes of *S. maliger* and *S. norvegicus* were between 16,401 and 17,609 bp, consistent with studies showing that the mtDNA control region of *Sebastes* is highly variable due to repetitive DNA sequences (Zhang et al. 2013; Sandel et al. 2018). The mitogenomes consist of 13 protein-coding genes, 22 tRNAs, two rRNAs, and one control region, and the order is identical to published *Sebastes* mitogenomes (Kim and Lee 2004; Zhang et al. 2013; Jang et al. 2016; Oh et al. 2016; Sandel et al. 2018; Kim et al. 2019). Twelve of the 13 protein coding genes had an ATG start codon; COX1 had a GTG start codon. Six genes (ND1, COX1, ATP8, ND4L, ND5, and ND6) had a TAA stop codon, three genes (ND2, ATP6, and COX3) had an incomplete TA stop codon, and four genes (COX2, ND3, ND4, and CytB) had an incomplete T stop codon.

Two phylogenetic trees were generated using W-IQ-Tree (Trifinopoulos et al. 2016) and BEAST 2.5 (Bouckaert et al 2019). We used *Sebasticus tertius* (MT117231) as an outgroup. Both phylogenetic analyses indicated that *S. maliger* is sister to a clade including *S. rubrivinctus, S. nigrocinctus, S. umbrosus,* and *S. oculatus*, and that *S. norvegicus* is sister to *S. fasciatus* (Figure 1). For the maximum likelihood phylogeny, all relationships are consistent with those previously published, except for *S. steindachneri*, which is sister to *S. minor* instead of *S. melanostictus* (Sandel et al. 2018; Kim et al. 2019), and *S. owstoni*, which is sister to *S. taczanowskii* instead of *S. minor* (Hyde and Vetter 2007). The Bayesian phylogeny (not shown) is identical to the maximum likelihood phylogeny except that *S. steindachneri* is sister to *S. melanostictus* instead of *S. minor,* and *S. nigrocinctus* is sister to *S. rubrivinctus* instead of *S. umbrosus* and *S. oculatus*. Several nodes in our maximum likelihood phylogeny had low bootstrap support (≤95) while all nodes in the Bayesian phylogeny had posterior probabilities >.99. Although additional species and analyses are needed to explore the differences seen in our phylogenies, the mitogenomes of *S. maliger* and *S. norvegicus* provide new insight into the phylogenetic relationships in *Sebastes*.

**Figure 1.**
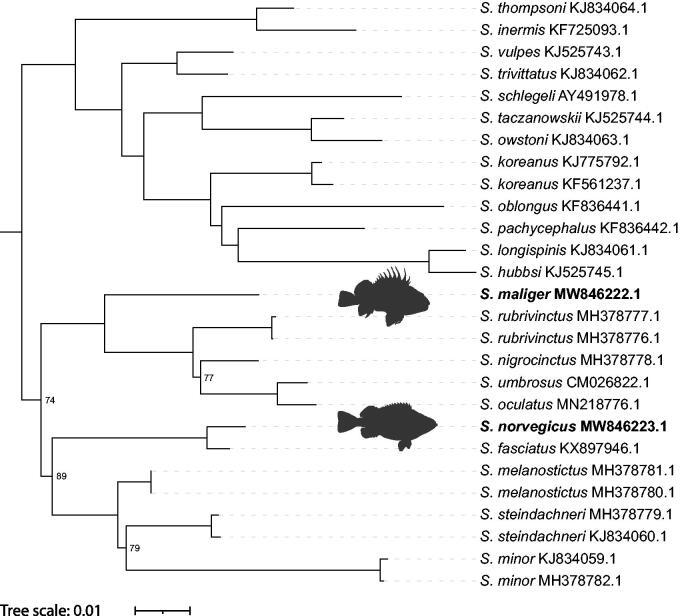
Phylogenetic tree inferred by maximum likelihood using W-IQ-Tree from 27 *Sebastes* mitogenomes. *Sebastiscus tertius* (MT117231) was used as an outgroup but is not displayed. Ultrafast bootstrap values >95 are not shown. “*Sebastes norvegicus*” by Jan Fekian is licensed under CC BY-SA 4.0/Silhouette of original.

## Ethical approval

*Sebastes maliger* was collected under Brigham Young Universities’ IACUC-approved protocol #15-0602 for DKS. *Sebastes norvegicus* was caught by commercial fisherman (Malmstrøm et al. 2017).

## Author contributions

The author contributions were as follows: **Jillian R. Campbell:** Analysis and Interpretation, Writing – Original Draft, Writing – Review and Editing., **Peter C. Searle:** Conception and Design, Analysis and Interpretation, Writing – Review and Editing. **Andrea L. Kokkonen:** Conception and Design, Analysis and Interpretation, Writing – Review and Editing. **Dennis K. Shiozawa:** Conception and Design, Analysis and Interpretation, Writing – Review and Editing. **Mark C. Belk:** Conception and Design, Analysis and Interpretation, Writing – Review and Editing. **R. Paul Evans:** Conception and Design, Analysis and Interpretation, Writing – Review and Editing. All authors approve the publication of this manuscript and agree to be accountable for all aspects of the work.

## Data Availability

The genome sequence data that support the findings of this study are publically available in GenBank of NCBI at (https://www.ncbi.nlm.nih.gov/) under accession numbers MW846222 and MW846223. The associated BioProject number for *S. maliger* and *S. norvegicus* is PRJNA741690, which holds a growing dataset of *Sebastes* mitogenomes. The associated BioSample numbers for *S. maliger* and *S. norvegicus* are SAMN20892470 and SAMEA4028819, respectively. The associated SRA numbers for *S. maliger* and *S. norvegicus* are SRR15573079 and ERR1473911, respectively.
